# What Do We Know About Component Separation Techniques for Abdominal Wall Hernia Repair?

**DOI:** 10.3389/fsurg.2018.00024

**Published:** 2018-03-27

**Authors:** Hubert Scheuerlein, Andreas Thiessen, Christine Schug-Pass, Ferdinand Köckerling

**Affiliations:** ^1^Department for General and Visceral Surgery, Vincenz Hospital, Paderborn, Germany; ^2^Department of Surgery and Center for Minimally Invasive Surgery, Vivantes Hospital, Academic Teaching Hospital of Charité Medical School, Berlin, Germany

**Keywords:** component separation technique, transversus abdominis release, endoscopic component separation technique, perforator sparing component separation technique, robotic transversus abdominis release

## Abstract

**Introduction:**

The component separation technique (CST) was introduced to abdominal wall reconstruction to treat large, complex hernias. It is very difficult to compare the published findings because of the vast number of technical modifications to CST as well as the heterogeneity of the patient population operated on with this technique.

**Material and Methods:**

The main focus of the literature search conducted up to August 2017 in Medline and PubMed was on publications reporting comparative findings as well as on systematic reviews in order to formulate statements regarding the various CSTs.

**Results:**

CST without mesh should no longer be performed because of too high recurrence rates. Open anterior CST has too high a surgical site occurrence rate and henceforth should only be conducted as endoscopic and perforator sparing anterior CST. Open posterior CST and posterior CST with transversus abdominis release (TAR) produce better results than open anterior CST. To date, no significant differences have been found between endoscopic anterior, perforator sparing anterior CST and posterior CST with transversus abdominis release. Robot-assisted posterior CST with TAR is the latest, very promising alternative. The systematic use of biologic meshes cannot be recommended for CST.

**Conclusion:**

CST should always be performed with mesh as endoscopic or perforator sparing anterior or posterior CST. Robot-assisted posterior CST with TAR is the latest development.

## Introduction

The component separation technique (CST) was introduced for abdominal wall reconstruction to treat large, complex hernias (1). The options for closing large and complex abdominal wall defects, including primary repair, mesh, and distant muscle flaps, have yielded suboptimal results (1). Albanese and Ramirez first developed the CST to address this issue (2–6). “CST is based on the concept of re-establishing a functional abdominal wall with autologous tissue repair” (1). “The procedure involves dividing the relatively fixed external oblique aponeurosis and muscle, elevating the rectus abdominis muscle from its posterior rectus sheath, and then mobilizing the myofascial flap consisting of the rectus, internal oblique, and transversus abdominis medially” (1).

In the meantime numerous different CSTs have been described. In the “classic” CST a distinction is made between the open anterior and posterior approach. In recent years a special type of posterior CST, transversus abdominis release (TAR), was introduced. Furthermore, endoscopic variants of the anterior and posterior CST have been developed. The most recent innovations are the laparoscopic and robot-assisted TAR.

By now, there are several studies and reviews that report on the CST range of topics. But this is hampered by the, in some cases fundamental, technical differences, widespread heterogeneity of the data, different methodological approaches to data evaluation and results assessment, and accordingly the overall large variance in the quality of the studies. The postulated advantages of CST are thought to derive especially from lower recurrence and morbidity rates. While at first glance these advantages appear plausible, to date it has not been possible to demonstrate any tangible benefit conferred by CST. On the contrary, wound infection rates of up to 57% have been identified (1).

The following review of the literature presents the different CST variants followed by a summary of the findings for each variant. The main focus here is on ascertaining the preferred CST variant based on the available data. Accordingly, particular attention will be paid to the findings of systematic reviews and combined literature sources.

## Systematic Literature Search

The literature search was carried out up to August 2017 using Medline (PubMed). CST related-topics were systematically searched using appropriate search terms (component/s separation, component separation technique, fascial component separation, separation of components, component release, separation of parts, complex ventral hernia repair, complex abdominal wall repair, giant hernia). In addition, a manual search of the references was performed to identify relevant publications. All types of clinical trials and systematic reviews were included at first.

Duplicate publications, when identified, were excluded. Likewise excluded were studies with *n* < 10 patients or with highly specific key questions which did not appear to lend themselves for literature comparison purposes (e.g., case reports, parastomal hernias, purely experimental studies, pediatric abdominal wall defects, studies with exclusively post-traumatic abdominal wall defects, CST performed in the setting of hyperthermic intraperitoneal chemotherapy (HIPEC) or CST conducted essentially in conjunction with flap surgery).

## CST Modifications

Using as search term “CST”, numerous different techniques can be found in the literature. However, direct comparison is hampered by the, in some cases fundamental, anatomical differences. The current literature does not permit any clear statement to be made regarding a particular CST type. CST types can be distinguished on the basis of the following criteria: on the one hand, anterior versus posterior CST and, on the other hand, open versus minimally invasive techniques. Ramirez et al. (4) demonstrated in anatomical studies “that the external oblique muscle can be separated from the internal oblique muscle in a relatively avascular plane”. The rectus muscle with its overlying rectus fascia can be elevated from the posterior rectus sheath. The compound flap of the rectus muscle, with its attached internal oblique and transversus abdominis muscles, can be advanced 10 cm around the waistline. The external oblique has limited advancement.

*“classic” anterior component separation technique* is well described by Clarke (7):

“Midline scar excision is followed by extensive skin flap mobilization. The lateral border of the rectus muscle is located, as well as a point 1 cm lateral to the rectus, the external oblique aponeurosis and muscle are divided from the inguinal region to the costal margin. Lateral dissection deep to the external oblique allows creation of a “sliding myofascial flap” consisting of internal oblique and transversus muscles. Cephalad to the costal margin, where the rib cage protects against herniation, the lateral border of the rectus may be released to allow these muscles to be mobilized from the chest wall and apposed in the midline to “fill” the epigastrium. Attenuated tissue around the hernia is resected, and the posterior rectus sheath may also be incised longitudinally, if additional mobilization is desirable. The midline is then closed with a single layer of heavy monofilament suture” (7).

Carbonell et al. (8) have published the technique of *“classic” posterior component separation”*:

“A midline laparotomy is performed with complete lysis of adhesions. Retromuscular space is developed by incising the posterior rectus sheath and dissecting the rectus muscle anteriorly. Once the lateralmost edge of the rectus sheath is reached, the posterior rectus sheath is incised, dividing the posterior aponeurotic sheath of the internal oblique muscle. This allows access to the plane between the internal oblique and transversus abdominis muscle. Dissection is carried out as far lateral, inferior, and superior as desired, allowing for a large mesh underlay. The posterior rectus sheath is then reapproximated in the midline with a running suture. The mesh is placed in the retromuscular space and secured with sutures. The anterior rectus sheath is then reapproximated in the midline to cover the mesh” (8).

**Figure 1:**
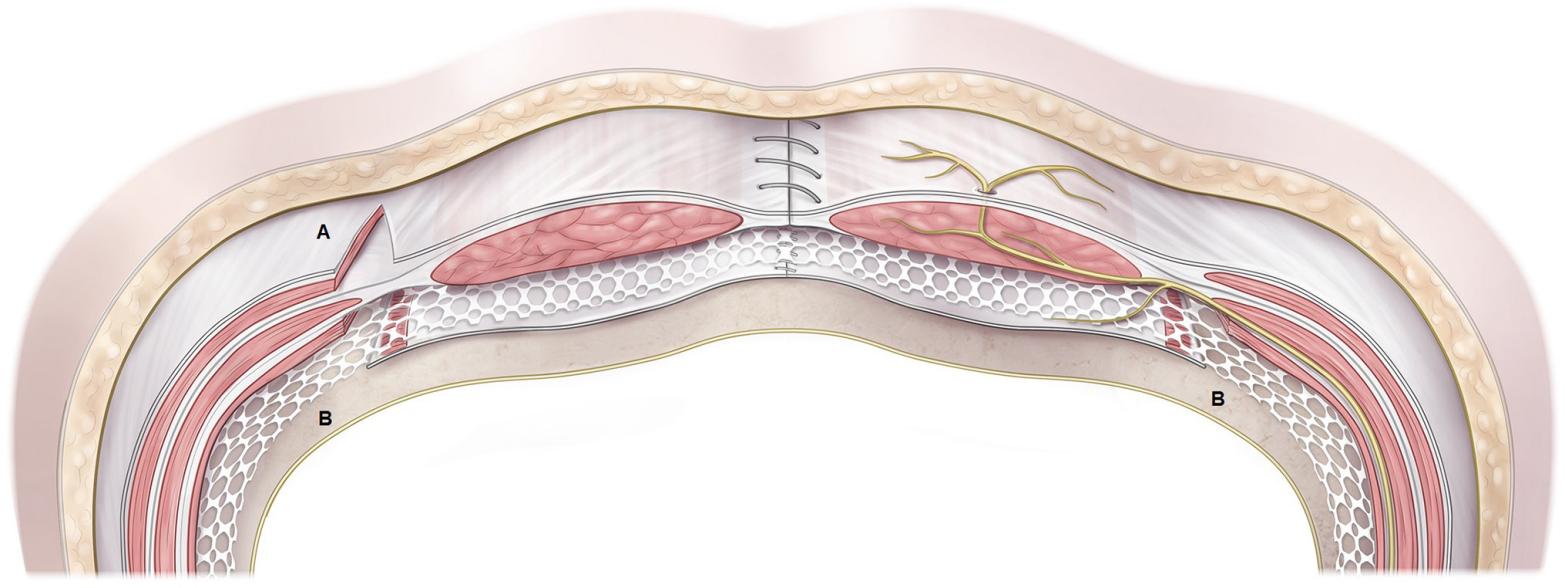
Schematic drawing of endoscopic component separation technique **(A)** and transversus abdominis release **(B)**.

Novitsky et al. (9) have described the novel technique of *transversus abdominis release (TAR)* (Figure 1B):

“The retromuscular plane is developed toward the linea similunaris, visualizing the junction between the posterior and anterior rectus sheaths. The perforators to the rectus muscle (branches of the thoracoabdominal nerves, penetrating the lateral edge of the posterior rectus sheath) are visualized and preserved. Starting in the upper third of the abdomen, about 0.5 cm medial to the anterior/posterior rectus sheath junction, the posterior rectus sheath is incised to expose the underlying transversus abdominis muscle. The muscle is then divided along its entire medial edge using electrocautery. This step is initiated in the upper third of the abdomen where medial fibers of the transversus abdominis muscle are easiest to identify and separate from the underlying fascia. This step allows entrance to the space between the transversalis fascia and the divided transversus abdominis muscle. Once similar release is performed on both sides, the posterior rectus sheaths are reapproximated in the midline with a running monofilament suture. The mesh is placed as a sublay in the retromuscular space and secured with sutures. The anterior rectus sheaths are then reapproximated in the midline to restore the linea alba ventral to the mesh”(9).

Clarke (7) has also reported about the details of the *anterior component separation technique with perforator preservation using balloon dissection*:

“Fascial separation is done through separate inguinal incisions. After incising the external oblique aponeurosis as in standard inguinal hernia repair, the balloon dissector is placed between the external and internal oblique muscles, advanced cephalad, and inflated . The lateral border of the rectus muscle acts as an anatomical barrier and forces the balloon to expand laterally, creating the necessary space. With headlamp illumination and a narrow retractor, a sponge forceps completes the fascial separation . Ultrasonic shears are then used to incise the elevated external oblique aponeurosis and the muscular portion found more cephalad. After fascial release has been done bilaterally, the midline scar is excised and minimal skin flaps are raised to free the hernia sac, thus preserving the periumbilical perforator vessels. The posterior rectus sheath is incised from within the midline incision. Midline closure is done as described for the “classic” anterior CST” (7).

Rosen et al. (10) have reported their initial experience with the *endoscopic anterior component separation technique of parts* (Figure 1A):

“The operation begins with a 1 cm incision just below the costal margin lateral to the rectus abdominis muscle. The subcutaneous tissues are bluntly divided, exposing the external oblique aponeurosis. The fibers are split in their natural orientation and the internal oblique muscle is exposed. The potential space between the internal and external oblique is created using a bilateral endoscopic inguinal hernia balloon dissector. A structural balloon port is then placed in this space to maintain insufflation pressures of 12 mmHg. The tip of a 10 mm 30° laparoscope is utilized to bluntly dissect the space under direct vision. Two additional 5 mm ports are then placed. The external oblique is then released from the costal margin to the inguinal ligament using coagulating scissors or ultrasonic shears. The process is repeated on the opposite side. If additional release is deemed necessary, the posterior rectus sheath is incised from the midline wound” (10).

Belyansky et al. (11) have presented the novel technique of *laparoscopic posterior component separation technique and transversus abdominis release*:

“Typically three ports are placed bilaterally. After reduction of the hernia content and adhesiolysis, the posterior rectus sheath is released approximately 0.5 to 1 cm lateral to the edge of the defect and linea alba. The incision in the posterior rectus sheath is performed along its whole length from cephalad to caudal direction. Hook electrocautery combined with laparoscopic scissors sharp dissection are used to achieve this release, which exposes the posterior portion of the rectus abdominis muscle. When performed appropriately it can allow up to 3 cm of medial mobilization of the edge of the defect. Once the posterior rectus sheath is released, atraumatic graspers are used to retract the free edge of posterior rectus sheath medially to facilitate blunt dissection in the retrorectus space laterally toward the linea semilunaris. The neurovascular bundles that travel between the internal oblique and transversus muscles and then perforate the rectus abdominis muscle are identified laterally in this space and are preserved to prevent rectus defunctionalization and atrophy. With preservation of the neurovascular bundle about 0.5–1 cm medial to the anterior/posterior rectus sheath junction, the posterior rectus sheath is incised to expose the underlying transversus abdominis muscle. Hook electrocautery is then used to elevate the transversalis muscle fibers and cauterize them. As the transversus fibers are cut the posterior layer of glistening transversalis fascia is exposed. Transversalis release is performed in this fashion in a cephalad to caudal direction. Blunt dissection just posterior to the transversus muscle and superficial to the transversalis fascia is performed and is carried past the midaxillary line. Unilateral TAR can achieve up to 7 cm of fascial medial mobilization. After closure of the posterior rectus sheath in a running fashion, the mesh is placed behind the muscles and fixed. The anterior fascial defect is then sutured “upside down” (11).

The use of *robotic surgery* to perform the *transversus abdominis release* technique has also been reported meanwhile (12).

### Should the “Classic” CST (Anterior Open) Without Mesh Be Performed?

“Among patients with midline abdominal incisional hernias, mesh repair is superior to suture repair with regard to the recurrence of hernia, regardless of the size of the hernia” (13, 14). As repair of ventral hernias with mesh has substantially improved long-term outcomes, it is therefore accepted as the standard of care (15, 16). Against that background the question arises as to whether the CST without mesh as reported by Ramirez (4) can be performed with acceptable results. In a review article Tong et al. (17) compared open CST with and without mesh. Patients who had open CST with mesh appeared to do better than those who had open CST alone. Open CST with mesh compared with open CST alone appeared to be associated with fewer hernia recurrences with mesh (16.7%) vs without mesh (27%) and overall complications (with mesh: 21% vs without mesh: 59%) (17).

A systematic review by Deerenberg et al. (18) found seven studies of incisional hernia repair with the “classic” CST, including one randomized controlled trial. A total of 219 large incisional hernias were repaired using CST without mesh. In approximately 40% of cases, patients had a complex incisional hernia. Postoperative complications occurred in almost 50%. Infection or necrosis of the wound occurred in 20%, hematoma in 8% and seroma in 9%. Recurrence after CST occurred in 16% (18).

In a systematic review of autologous tissue repair of large abdominal wall defects, CST was the best documented procedure (19). It was associated with a high morbidity rate of 24.0%. After a follow-up period of at least one year the recurrence rate was 18.2% (19).

In a qualitative systematic review for treatment of giant incisional hernias the authors concluded that mesh repair appeared to be superior to CST without mesh with regard to the recurrence rate (20). In open CST without mesh outcomes were beset with high morbidity, related mainly to major wound complications, skin necrosis, hematoma, pulmonary complications (23–100%) and a high clinical recurrence rate (5–53%) within a follow-up period ranging from 15 to 52 months (20).

Slater et al. (21) performed their own prospective study and compared their results with a literature review. Prospective patient follow-up was undertaken of consecutive patients who underwent repair of large and complex ventral hernias using CST without mesh utilization (21). Primary outcome was recurrent hernia determined by clinical examination at least one year after surgery. Meta-analysis of the current literature was performed regarding outcomes and mode of follow-up. This included 75 patients with a mean age of 52.2 years and mean defect size of 214.9 cm^2^. Twenty-nine patients (38.7%) had a recurrent hernia after a mean of 40.9 month follow-up, and this was significantly higher than in the literature (14.0%, *p* < 0.01). Sixty-four per cent of studies in the literature were unclear about the method of determining recurrent hernia or included telephone follow-up and questionnaires (21). The authors concluded that repair of large ventral hernias using CST without mesh coincides with a high recurrence rate (21).

In the only prospective randomized study comparing CST without mesh vs mesh repair in giant midline abdominal wall hernia, the recurrence rate in the CST group without mesh after 36 months was 52.6% (22).

In a case series of 85 patients with repair of complex abdominal wall hernias using CST, the overall recurrence rate after a mean follow-up of 14.4 months was 14.1 and 11.1% when a mesh was used to reinforce the repair technique (23).

In conclusion, the hernia recurrence rates remain high without mesh reinforcement even when using tension-reducing procedures such as CST (24) and should therefore no longer be performed. In an expert consensus guided by systematic review of ventral hernia management, the avoidance of CST without mesh reinforcement is therefore recommended (24).

### Do Perioperative Complication Rates Justify Performance of “Classic” (Anterior Open Without Mesh) or Modified (Anterior Open with Mesh) CST?

Unfortunately, standard anterior CST is not without its own procedural morbidity (1). The extensive lateral dissection required to create large subcutaneous skin flaps leads to marked wound complications (1). “Specifically, ligating a significant proportion of the perforating abdominal wall blood vessels predisposes the flap to ischemia and infection, in addition to potential formation of hematomas and seromas in the dead space (1). Wound infection rates have been shown to range from 25 to 57%” (1).

In the review by Tong et al. (17) the overall complication rate was reported to be 21% with mesh and 50% without mesh. In their review Deerenberg et al. (18) identified for anterior open CST without mesh a postoperative complication rate of almost 50% and for anterior open CST with mesh of 55%.

In the review of surgical treatment for giant incisional hernias by Eriksson et al. (20), the total morbidity following CST with or without mesh ranged between 15 and 100%.

In the only randomized controlled trial the wound complication rate after “classic” anterior open CST without mesh was reported to be 52.6% (22).

Against a background of the high complication rate associated with the “classic” and modified anterior open CST, the guidelines recommended performance of the alternative CST, such as a perforator sparing, endoscopic or posterior CST technique (16, 24).

### Does Perforator Sparing or Endoscopic Anterior CST Have Advantages Compared to “Classic” or Modified Anterior Open Techniques?

In a retrospective comparative study Clarke (7) reported significantly higher rates of skin necrosis (*p* < 0.001) and chronic pain (*p* = 0.003) for the “classic” technique vs the perforator preservation procedure with fascial release through separate inferolateral incisions.

In a review Pauli et al. (25) showed for perforator sparing CST a wound complication rate of 3.1%-26.3% and a recurrence rate of 3–13.8%.

Switzer et al. (1) have published a systematic review and meta-analysis with inclusion of 63 primary studies (seven controlled studies and 56 cases series) and 3,055 patients comparing endoscopic anterior vs open CST. “The total wound complication rate was lower for endoscopic anterior CST (20.6%) compared to open CST (34.6%). Endoscopic anterior CST compared to open CST was shown to have lower rates of superficial infections (3.5 vs 8.9%), skin dehiscence (5.3 vs 8.2%), necrosis (2.1 vs 6.8%), hematoma/seroma formation (4.6 vs 7.4%), fistula tract formation (0.4 vs 1.0%), fascial dehiscence (0 vs 0.4%), and mortality (0.4 vs 0.6%). The open CST did have lower rates of intraabdominal abscess formation (3.8 vs 4.6%) and recurrence rates (11.1 vs 15.1%)” (1). Four further studies not included in the systematic review come to the same conclusions (26–29).

A recent systematic review of CST for giant incisional hernia reported for open anterior CST and endoscopic anterior CST surgical site occurrences of 21.4 and 20.3%, respectively, and recurrence rates of 11.9 and 7%, respectively (30).

The guidelines recommend that preference be given to the endoscopic anterior CST over the “classic” or modified anterior open CST (24).

## Does Posterior CST Have Better Results Than Anterior CST?

Retromuscular or sublay hernia repair with mesh ”has proven to be a durable technique for ventral hernia defects, and completely avoids subcutaneous flap elevation” (8). “Technically, the retromuscular technique requires developing the space dorsal to the rectus abdominis muscles up to the edge of the rectus sheath” (8). “In the average patient, this translates into a 6–8 cm lateral space on each side of the midline” (8). “Repair of large hernia defects with diameters greater than 15 cm may require a larger mesh overlap than can be afforded by dissection limited to the confines of the rectus sheath” (8). “By incising the posterior rectus sheath and creating the plane between the internal oblique and transversus abdominis muscles (“classic” posterior CST), there is a space virtually unlimited in size in which to place large meshes for hernia repair” (8).

In a case series of 20 patients with a “classic” posterior CST three developed wound complications (15%) and none complained of long-term pain or abdominal wall deformity (8). There has been one recurrence (5%) after a mean 12 month follow-up (8).

Novitsky and Rosen (9) developed a novel technique of posterior component separation using transversus abdominis muscle release (TAR). This modification allows for significant posterior rectus fascia advancement, wide lateral dissection, preservation of the neurovascular supply, avoids subcutaneous tissue undermining, and provides a large space for mesh sublay (9). In the case series by Novitsky and Rosen (9) the TAR technique was used in 42 patients with complex ventral hernia, including 32 recurrences, with an average number of previous repairs of 2.9 (range, 1–8). Postoperative wound complications occurred in 10 (24%) patients with three (7%) major wound infections (9). At a median follow-up period of 26.1 months, there have been two (4.7%) recurrences (9).

In a review by Pauli et al. (25) the outcome for posterior component separation including TAR, showed a wound complication rate of 3.4–31% and a recurrence rate of 1.1–7.3%.

In a retrospective comparative study by Krpata et al. (31) wound complications occurred in significantly more open anterior CST than open posterior CST (48.2 vs 25.5%, *p* = 0.01). The recurrence rate was also higher in the open anterior CST group (14.3 vs 3.6%, *p* = 0.09) (31).

Posterior component separation with transversus abdominis release also successfully addresses recurrent ventral hernia following anterior component separation (32).

For complex incisional hernias in kidney transplant recipients, TAR is associated with low perioperative morbidity and durable repair (33).

In the largest case series of 428 consecutive TAR procedures 80 (18.7%) surgical site events occurred, of which 39 (9.1%) were surgical site infections. Three patients required mesh debridement; however, no instance of mesh explantation occurred. Of the 347 (81%) patients with at least one-year follow-up (mean 31.5 months), there were 13 (3.7%) recurrences (34).

The recent systematic review by Cornette et al. (30) included 22 studies with 1,348 cases for open anterior approach and eight studies with 761 cases for posterior CST with transversus abdominis release (TAR). They found surgical site occurrence rates of 21.4% for the open anterior approach and 20.3% for posterior CST with TAR. The recurrence rates were 11.9 vs 5.25% (*p* < 0.001) (30).

The guidelines of an expert group contain a statement that posterior CST is associated with a lower wound complication rate in comparison to standard anterior CST (24). However, the findings of the systematic review tend to show advantages for posterior CST with TAR with regard to the recurrence rate rather than to surgical site occurrences (30).

### Are There Differences Between the Perforator Sparing Anterior, Endoscopic Anterior and Posterior CST?

While there are no prospective randomized trials or meta-analyses, the available data demonstrate that preference should be given to the perforator sparing anterior, endoscopic anterior and “classic” or TAR posterior CST over the “classic” and modified anterior CST. A mesh should always be used for CST. Among these remaining CST cases there is no consensus in the guidelines of an expert group on the best CST technique because of a lack of high quality data (24).

The best comparative analysis can be found in the recently published systematic review by Cornette et al. (30). In addition to eight studies and 761 cases related to TAR, this included 13 studies with 193 cases for endoscopic anterior CST and five studies with 242 cases for anterior perforator sparing CST. The identified surgical site occurrence rates were 16.0% for perforator sparing anterior CST, 20.3% for endoscopic anterior CST, and 23.7% for TAR. The corresponding recurrence rates were 6.47, 7.02 and 5.25%, respectively. Based on that systematic review, no relevant differences were identified between these techniques.

This serves as confirmation of the statement issued by the expert committee on that comparison (24). Accordingly, high quality studies are urgently needed to explore this key question.

## Robotic Posterior CST with Transversus Abdominis Release (TAR)

The introduction of posterior CST with transversus abdominis release using a robot-assisted technique is the latest development in the CST setting (12). Warren et al. (34) compared 103 standard laparoscopic ventral hernia repairs with 53 robotic posterior CST with transversus abdominis release (Table 1). The hernia width was similar in both groups (6.9 vs 6.5 cm, *p* = 0.508). Fascial closure was achieved more often with the robotic technique (96.2 vs 50.5%; *p* < 0.001). But the operative time was longer with the robot (245 min vs 122 min; *p* < 0.001). Seroma was more common after robotic posterior CST with TAR (47.2 vs 16.5%; *p* < 0.001), but surgical site infection was similar (3.8 vs 1%; *p* = 0.592). The median length of stay was shorter after robot-assisted CST with TAR (1 vs 2 days; *p* = 0.004).

**Table 1:** Results of robotic transversus abdominis release and retromuscular repair compared to laparoscopic or open repair.

**Author**	**Robotic CTS**	**Technique for comparision**	**Hernia defect width or defect size**	**Fascial closure**	**Operative time**	**Morbidity**	**Seroma rate**	**SSI**	**Lenght of stay**	**Readmission rate**
Warren (35)	Robotic TAR *n* = 53	Standard laparoscopic repair *n* = 103	Mean 6.5 vs 6.9 cm width; ns	96.2 vs 50.5%; *p* < 0.001	Mean 245 vs 122 min; *p* < 0.001	Not specified	47.2 vs 16.5%; *p* < 0.001	3.8 vs 1.0%; ns	Mean 1 vs 2 days; *p* = 0.004	7.5 vs 4.8%; ns
Bittner (36)	Robotic TAR *n* = 26	Open TAR *n* = 76	Mean 260 vs 235 cm²; ns	100 vs 100%; ns	Mean 365 vs 287 min; *p* < 0.01	19.2 vs 39.4%; *p* = 0.09	Not specified	3.8 vs 2.6%; ns	Mean 6.7 vs 3.5 days; *p* < 0.01	7.7 vs 6.6%; ns
Martin-del- Campo (37)	Robotic TAR *n* = 38	Open TAR *n* = 76	Not specified	Not specified	Mean 299 vs 211 min; *p* < 0.001	0 vs 17.1%; *p* = 0.007	Not specified	0 vs 6.6%; ns	Mean 1.3 vs 6 days; *p* < 0.001	0 vs 2,63%; ns
Carbonell (38)	Robotic retromuscular ventral hernia repair *n* = 111	Open retromuscular ventral hernia repair *n* = 222	Mean 7.51 vs 7.17 cm width; ns	100 vs 99%; ns	Significantly longer for robotic repair (*p* < 0.001)	Not specified	25,2 vs 4,1%; *p* < 0,001	2 vs 4%; ns	Mean 2 vs 3 days; *p* < 0.001	6 vs 5%; ns

Bittner et al. (36) compared 76 open posterior CST with TAR and 26 robot-assisted posterior CST with TAR. Patients were comparable regarding age, gender, body mass index, and the presence of comorbidities. Diabetes was more common in the open group (22.3 vs 0%; *p* = 0.001). Most ventral hernias were midline (89.5 vs 83%; *p* = 0.47) and recurrent hernias (52.6 vs 58.3%; *p* = 0.65). Hernia characteristics were similar regarding defect size (260 ± 209 vs 235 ± 107 cm^2^; *p* = 0.55). The average operative time was longer in the robot cohort (287 ± 121 vs 365 ± 78 min; *p* < 0.01). Robot posterior CST with TAR trended toward lower morbidity (39.2 vs 19.2%; *p* = 0.09), less severe complications, and similar rates of surgical site events and readmission (6.6 vs 7.7%; *p* = 1.00). In addition, robotic posterior CST with TAR resulted in a significantly shorter median hospital length of stay compared to the open procedure (6 days, 95% CI 5.9–8.3 vs 3 days, 95% CI 3.2–4.3).

Martin-del-Campo et al. (37) compared 38 consecutive patients undergoing robotic posterior CST with TAR to 76 matched open posterior CST with TAR. Patient demographics were similar between the groups. The average operative time was significantly longer in the robotic TAR group (299 ± 95 vs 211 ± 63 min, *p* < 0.001) and blood loss was significantly lower for the robotic TAR group (49 ± 60 vs 139 ± 149 mL, *p* < 0.001). Wound morbidity was minimal in the robotic TAR group, but the rate of surgical site events and surgical site infection did not differ between the groups. Systemic complications were significantly less frequent in the robotic TAR group (0 vs 17.1%, *p* = 0.026). The length of hospitalization was significantly reduced in the robotic TAR group (1.3 ± 1.3 vs 6.0 ± 3.4 days, *p* < 0.001).

In a matched pair analysis, Carbonell et al. (38) compared 111 robot-assisted retromuscular ventral hernia repairs with 222 open retromuscular ventral hernia repairs from the Americas Hernia Society Quality Collaborative Database (AHSQC). Median length of stay was significantly decreased for robotic retromuscular ventral hernia repair (2 days vs 3 days, *p* < 0.001). No difference was observed in 30 day readmissions or surgical site infection. Higher surgical site occurrences were noted with the robotic technique, consisting mainly of seroma that did not require intervention.

From the comparative investigations conducted to date it can be concluded that the use of a robot results in a prolonged operative time but a shorter hospital stay. So far no clear evidence has been found of advantages in terms of wound complications. The robot technique also appears to be associated with a higher seroma rate.

Once sufficient experience has been gathered with robot-assisted posterior CST with TAR, the role of this technique compared with perforator sparing anterior, endoscopic anterior and open posterior CST with TAR must be ascertained in future comparative studies. With greater experience leading to a shorter operative time and lower robot costs per use, robot-assisted CST with TAR could become a mainstay for repair of complex abdominal wall hernias.

## Does the Use of Biologic vs Synthetic Meshes in CST Lead to a Better Outcome in Repair of Complex Abdominal Wall Hernias?

In a review by the BioMesh Study Group of the evidence available on the use of biologic meshes in abdominal wall reconstruction, studies that compared complex abdominal wall hernia repair with CST using biologic vs synthetic meshes were analyzed (39). Based on only comparative studies, CST reinforced with biologic meshes has no significantly higher recurrence rate vs synthetic meshes in patients with complex abdominal wall hernia repair and at increased risk for surgical site occurrences/surgical site infections (39). The number of major surgical site occurrences/surgical site infections, including the need for reoperation, does not seem to be decreased substantially by the use of biologic vs synthetic meshes (39). Therefore, the systematic use of biologic meshes in complex abdominal wall hernia repair in the absence of contamination is not recommended (39).

## Conclusions

- The “classic” anterior CST without mesh and “modified” anterior CST with mesh should no longer be performed because of too high surgical site occurrence rates. Besides, the “classic” anterior CST without mesh is associated with too high a recurrence rate.
- Endoscopic and perforator sparing anterior CST are better alternatives to anterior CST.
- Likewise, the “classic” posterior CST and posterior CST with transversus abdominis release produce better findings than the “classic” and “modified” anterior CST.
- Based on the data available so far, no significant differences can be identified between the findings obtained for endoscopic anterior CST, perforator sparing anterior CST and posterior CST with transversus abdominis release.
- At present, it is not possible to evaluate the role of laparoscopic and robot-assisted posterior CST with TAR.
- The systematic use of biologic meshes in CST for complex abdominal hernia repair in the absence of contamination is not recommended.
- The introduction of robot-assisted posterior CST with TAR is the latest development. While the findings so far reveal a prolonged operative time for the robot technique, they also show a shorter length of stay. To date, no conclusive findings are available regarding the perioperative complications.

## Author Contributions

HS: literature search, literature analysis, manuscript conception, and writing the manuscript. AT: literature search, literature analysis, and critical review of the manuscript. CS: literature search, literature analysis, and critical review of the manuscript. FK: literature search, literature analysis, concept of manuscript, and writing the manuscript.

## Conflict of Interest Statement

The authors declare that the research was conducted in the absence of any commercial or financial relationships that could be construed as a potential conflict of interest.
